# InertialMov: Machine Learning Test Based on Inertial Sensors to Predict Mobility Impairment in Low Back Pain Patients

**DOI:** 10.3390/s25216665

**Published:** 2025-11-01

**Authors:** Jeremy Carlosama, Luis Zhinin-Vera, Cesar Guevara, Carolina Cadena-Morejón, Diego Almeida-Galárraga, Lenin Ramírez-Cando, Kevin R. Landázuri, Andrés Tirado-Espín, Patricia Acosta-Vargas, Fernando Villalba-Meneses

**Affiliations:** 1 School of Biological Sciences and Engineering, Yachay Tech University, Urcuquí 100119, Ecuador; jeremy.carlosama@yachaytech.edu.ec (J.C.); dalmeida@yachaytech.edu.ec (D.A.-G.); lramirez@yachaytech.edu.ec (L.R.-C.); klandazuri@yachaytech.edu.ec (K.R.L.); gvillalba@yachaytech.edu.ec (F.V.-M.); 2 LoUISE Research Group, University of Castilla-La Mancha, 02005 Albacete, Spain; luis.zhinin@uclm.es; 3 Quantitative Methods Department, CUNEF Universidad, 28040 Madrid, Spain; cesar.guevara@cunef.edu; 4 School of Mathematical and Computational Science, Yachay Tech University, Urcuquí 100119, Ecuador; ccadena@yachaytech.edu.ec (C.C.-M.); ctirado@yachaytech.edu.ec (A.T.-E.); 5 Intelligent and Interactive Systems Laboratory, Universidad de Las Américas, Quito 170125, Ecuador

**Keywords:** low back pain, Machine learning, inertial sensors, predictive models, ANOVA, regression models, clinical evaluation

## Abstract

Low back pain (LBP) is one of the leading causes of disability in the world's population, yet there are limitations in providing an objective clinical assessment due to its widespread nature. In this work, five machine learning models (LightGBM, XGBoost, HistGradientBoosting, GradientBoosting, and StackingRegressor) were compared to predict trunk mobility based on inertial sensor data. There were 77 individuals with a total of 2160 movement samples of flexion–extension, rotation, and lateralization. Synthetic data augmentation and normalization were performed to be able to work with the data efficiently. Mean absolute error (MAE), mean square error (MSE), and R2 were used to evaluate model performance. Additionally, ANOVA and Tukey’s HSD were used to assess the statistical significance of the models. GradientBoostingRegressor was found to produce the lowest error and statistical significance in flexion–extension and lateralization, while StackingRegressor produced the best error in rotation. The results indicate that inertial sensors and machine learning (ML) can be applied to predict mobility, facilitating personalized rehabilitation and reducing costs. The present study demonstrates that predictive trunk motion modeling can facilitate clinical monitoring and help reduce socioeconomic limitations in patients.

## 1. Introduction

Low back pain (LBP) is one of the leading causes of disability worldwide and has a significant socioeconomic impact in terms of healthcare costs and productivity [1]. Low back pain is pain located in the lower back, whose origin is related to the musculoskeletal structure of the spine. It is defined as muscle pain in the lower back (L1–L5), causing increased muscle tone and stiffness, and is one of the most common pathologies affecting people worldwide [2,3]. It is a multifactorial condition, often resulting from complex interactions between anatomical, biomechanical, and physiological factors. Low back pain can be classified into two main categories: specific, which refers to cases where there is a clearly identifiable cause, such as herniated discs, spinal stenosis, or vertebral fractures [4], and nonspecific, which represents the majority of cases, lacks an identifiable anatomical cause, and is frequently associated with soft tissue injuries or age-related degenerative changes in spinal structures [5,6].

In Ecuador, the rising rate of LBP, especially in middle-aged people, has highlighted the need for new diagnostic techniques and more effective treatments to ease the burden on medical services. The prevalence has steadily increased in recent years, leading to higher rates of work disability and increased demand for chronic pain treatment [7,8]. According to a study by Fuseau et al. ([9]), between 2017 and 2020, 2055 clinical consultations for LBP were recorded in a health center in Imbabura, the majority of which corresponded to women between 28 and 60 years of age. Unfortunately, no apparent decrease in incidence was observed in those over 60 years of age, as reported in international studies. The costs of low back pain (LBP) are further increased by the complexity of its diagnosis and treatment due to its inadequacy in cases of nonspecific LBP, where diagnostic tools, such as imaging, are ineffective in localizing the source of the pain [10]. The current common diagnostic methods combine physical examination and imaging tests, which can be a source of valuable clinical information, but which, in most cases, fail to diagnose the origin of nonspecific LBP, as the underlying causes remain unidentified [11,12]. This deficiency in diagnosis is fundamental, since LBP remains one of the leading causes of disability in the world, with a substantial impact on healthcare systems, the quality of life of those affected, and the productivity levels of our societies [13].

The recent development of wearable inertial measurement units (IMUs) and the advancement of machine learning (ML) have facilitated more affordable motion analysis. Previous studies using IMUs to classify LBP conditions with the help of ML have shown promising results, but have mainly focused on diagnostic classification rather than predicting current quantitative measures of movement directly relevant to rehabilitation [14,15]. It is essential to address this deficiency, as accurate measurement of trunk mobility will allow the therapist to individualize a treatment plan, as well as monitor rehabilitation progress for patients with LBP. In this regard, advanced technology-based methodologies emerge as a promising alternative, as conventional clinical tools cannot accurately identify pain sources in nonspecific cases [16]. It has recently been shown that the combination of machine learning (ML) algorithms and inertial measurement units (IMUs) can facilitate the prediction of patient mobility and increase diagnostic accuracy [17]. Reconstructing patients’ data of interest with these tools not only facilitates more valid assessments but also allows defining individual therapeutic approaches, which are essential for optimizing rehabilitation processes [18,19]. This step will contribute to improving the patient experience by optimizing clinical decision-making, resulting in better outcomes and a reduction in the economic and social cost of LBP in a low-resource care setting.

Recent research has increasingly combined inertial sensors with machine learning (ML) to improve low back pain (LBP) assessment, some of them are showed in Table 1. For example, Kraus et al. (2023) [20] used Random Forest classifiers with IMU data to distinguish LBP patients from healthy controls, while Hartley et al. (2024) [21] applied SVMs with accelerometer data to detect abnormal trunk movements. Similarly, Molnar et al. (2018) [22] leveraged convolutional neural networks with lumbar-mounted IMUs to identify impaired mobility levels. Although these approaches demonstrate the feasibility of sensor-based ML for LBP analysis, they primarily address classification tasks rather than the prediction of continuous mobility metrics directly applicable to rehabilitation. Other studies have explored ensemble approaches. Salvatore et al. (2023) [23] used Gradient Boosting to estimate trunk range of motion, Camargo et al. (2021) [24] implemented XGBoost to classify postural patterns, and Pouromran et al. (2021) [25] predicted functional rehabilitation scores using ensemble machine learning (ML). While these contributions highlight the potential of ensemble methods for musculoskeletal assessment, they often lack comparative evaluations across multiple models or do not focus on specific trunk kinematics during rehabilitation. Additional studies (e.g., Keller et al., 2022 [26]; Laird et al., 2019 [27]) underline the need for objective mobility assessment but still rely on expensive motion capture systems or descriptive analytics. Taken together, the literature confirms the value of ML and inertial sensors, but reveals a gap: few studies predict quantitative trunk mobility metrics that support personalized rehabilitation. The present study addresses this issue by comparing five ensemble regressors to predict trunk velocity in flexion–extension, rotation, and lateralization tasks, providing an objective framework for monitoring individualized therapy.

This study advances biomedical engineering knowledge through the combination of contemporary technologies that address a significant clinical and social issue. For that, this project aims to address the growing need to improve treatment approaches for low back pain by investigating the use of machine learning algorithms in the analysis of relevant patient data. This will be achieved through supervised machine learning regression to forecast continuous trunk kinematic measures measured via wearable Inertial Measurement Units (IMUs) over the course of a structured rehabilitation program. Specifically, the models must project the per-motion characteristics of trunk kinematic features, measured via three IMUs at the forehead, C7, and sacrum, to the mean trunk angular velocity (Vel), which is the clinical outcome. The models enable longitudinal monitoring by predicting change in measured characteristics at earlier stages of the treatment sequence: consistent increases in predicted (and measured) Vel values during treatment sessions signal increased mobility and thus a beneficial treatment response. In this paper, a comparative study of five ensemble regressors, LightGBM, XGBoost, HistGradientBoostingRegressor, GradientBoostingRegressor, and a StackingRegressor, was performed using patient-level five-fold cross-validation. Performance was determined based on the mean absolute error (MAE), mean squared error (MSE), and coefficient of determination (R^2^). Additionally, pairwise statistical comparisons (ANOVA and Tukey’s honest significant difference (HSD) post hoc test) were performed to carefully estimate the most robust algorithms for use in clinical monitoring applications. Addressing this challenge is crucial to optimize short-term patient outcomes and reduce the social and economic burden of this widespread condition.

## 2. Materials and Methods

### 2.1. MoCap Dataset

The inertial sensor dataset used in this research is obtained from the MoCap database presented by Villalba et al. (2024) [8], which contains three populations: healthy controls, patients who had a routine physical convention, and patients who had deep oscillation therapy. For this study, we focused exclusively on the deep oscillation therapy group, as these patients underwent a structured rehabilitation protocol with repeated mobility assessments in three stages (before, during, and after therapy). This provided longitudinal and clinically relevant mobility data, making it the most suitable subgroup to evaluate the ability of machine learning models to predict trunk movement during rehabilitation. The full sample included 77 individuals in Imbabura, Ecuador (aged 18–65 years and of both sexes), composed of 40 pathological subjects with acute, subacute, or chronic low back pain and 38 asymptomatic controls. Exclusion criteria excluded individuals who had undergone physical or pharmacological therapy within the previous 6 months prior to data collection. Over a 6-month period, participants were encouraged to perform repeated axial exercises, which were processed using motion capture technology, entering 2160 samples from individuals during and after 12 deep oscillation therapy sessions using three functional movements:

- Flexion–extension: Starting in a standing position, the patient leans forward with their arms extended until they touch their toes. They then return to the initial standing position and then lean backward in the sagittal plane (Rx).
- Rotation: The patient should stand with their arms close to their chest and rotate their trunk from left to right in the transverse plane (Ry), keeping their waist fixed in position.
- Lateralization: In a standing position, the patient performs lateral movements with their back straight in the frontal plane (Rz).

All participants provided their informed consent in writing prior to participation, with information covering both the procedure and data management. The research protocol complied with ethical standards of human experimentation set by the Declaration of Helsinki and was approved by the institutional Ethics Committee of the Pontificia Universidad Catolica del Ecuador under approval number EO-146-2022. All data were anonymized by removing all personal identifiers before being analysed.

### 2.2. Technology and Instrumentation

**Figure 1:**
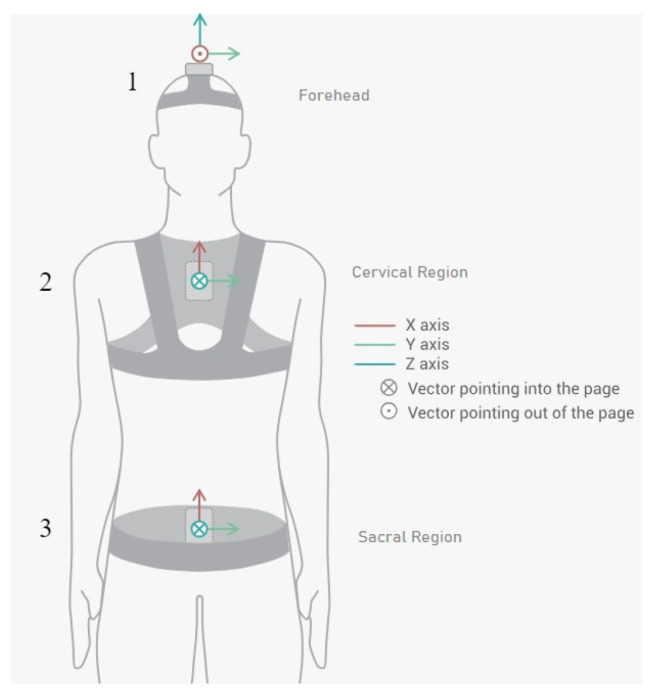
Inertial sensor placements and orientations on patient (sensor 1 in the forehead region, sensor 2 in the cervical region, and sensor 3 in the sacral region) [8].

The database of the research of Villalba et al. (2024) [8] was obtained using the Move Human (MH) Sensors MoCap system developed by IDERGO (V19-07.011, University of Zaragoza, Zaragoza, Spain) with NGIMU (x-io technologies, Bristol, UK). The use of this system relies on inertial measurement units (IMUs) securely placed on three important parts of the body: sensor 1, located on the top of the head (foreachad), sensor 2, located in the cervical region (at C7), and sensor 3, located in the sacral region (at the level of the iliac crest) (see Figure 1), to perform a comprehensive analysis. This system provides precise data on the rotations and displacements of the various body parts at a frequency of 60 Hz.

### 2.3. Data Preprocessing

A correlation analysis was conducted to identify the influential factors. The selection criteria focused exclusively on variables exhibiting strong positive or negative coefficients, thereby excluding metrics with weak or redundant correlations. Consequently, only the variables presented in Table 2 were considered for this study.

To increase robustness and generalizability across flexion–extension, rotation, and lateralization tasks, the dataset was supplemented with synthetically generated samples and rescaled to the 0–1 range to avoid the predominance of large magnitude features and stabilize convergence. Mean trunk velocity (Vel), the predictor variable of interest, was taken as the average angular velocity of the trunk throughout the different movements cycle. Angular velocity was obtained from the inertial sensor gyroscope and calculated in degrees per second (°/s). This variable is of great clinical relevance, as there is a correlation between lower trunk velocity and greater severity and functional limitation of low back pain in patients with low back pain [27], which constitutes an objective measure of mobility and functional disability.

To address the small sample size associated with the provided clinical mobility data, synthetic data were generated by introducing Gaussian noise (5% of the standard deviation of each feature) to simulate the natural heterogeneity of human movement as was studied by [28,29,30]. Augmenting this dataset with its statistical distribution resulted in a sevenfold increase, as confirmed by Kolmogorov-Smirnov tests. Noise-based amplification has been successfully applied to biomechanical and biomedical signals to improve model stability and limit overfitting. This strategy also allowed the trained models to be generalizable in terms of mobility patterns and not underfit the original data.

To minimize the risk of overfitting and data leakage, a strict 5-fold cross-validation strategy was applied. The dataset was partitioned at the patient level, ensuring that motion samples from the same individual were never included simultaneously in training and testing folds. This approach guaranteed independence between training and evaluation sets and provided a robust estimate of model generalization.

### 2.4. Predictive Models

This research used predictive models rather than classification to estimate continuous outcomes, specifically average movement velocity in people with low back pain. This will allow for an individualized approach to treatment and track patient progress with temporal biomechanical indicators. Various machine learning algorithms were applied to model flexion–extension movement patterns and accurately predict what might happen. These included LightGBM for its high speed and ability to resist outliers; XGBoost for improved regularization and memory usage; and HistGradientBoostingRegressor for reducing errors with high speed and large data volume. GradientBoostingRegressor was more stable in noisy situations, and StackingRegressor combined multiple base models, resulting in the best MAE and MSE, thanks to its greater generalization ability. The hyperparameters of the models were kept constant across all movements (flexion–extension, rotation, and lateralization) to ensure that the performance evaluation is a direct and fair comparison between the algorithms, minimizing the influence of parameter optimization on the results, explained in Table 3.

### 2.5. Visual Analysis Methods

Visual analysis can be important in complementing quantitative assessment with qualitative information about model behavior. These methods allow researchers to identify inconsistencies, detect systematic errors, and ensure that predictive models are highly generalizable when variations in value ranges occur. In biomedical applications, particularly in the case of low back pain, as presented in this article, visual tools facilitate the interpretation of accuracy and predictive ability, as well as decision-making through graphical analysis of data trends. To this end, scatter plots were used to show the proximity of predicted values to actual values and their analysis, and bar charts were used to show the range of values that exhibit errors and their analysis.

### 2.6. Statistical Analysis

A one-way analysis of variance (ANOVA) was used to assess differences between models in each of the three functional movements. If a statistically significant result was obtained, Tukey’s Honestly Significant Difference (HSD) post hoc test was used to compare differences between paired groups. All analyses were performed with a significance level of *p* < 0.05.

### 2.7. Study Workflow

**Figure 2:**
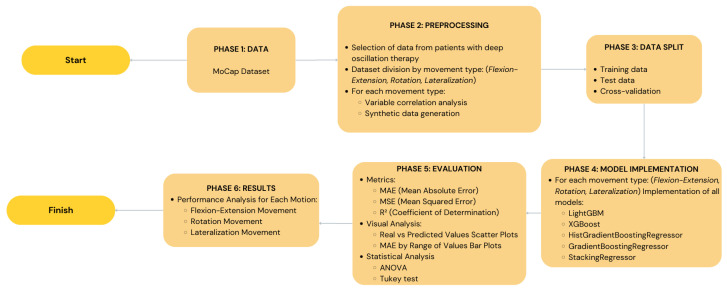
A diagram illustrating the entire study process, from data from inertial sensors to trunk mobility prediction using the five machine learning models. The preprocessing, data augmentation, and model performance evaluation stages are detailed.

In order to demonstrate the entire research process of our study including data collection and machine learning models testing, Figure 2 illustrates a schematic view of the research. This figure outlines all the steps such as the data collection, preprocessing, data augmentation, the use of the five machine learning models, and lastly the evaluation of the performance of the models. This graphical illustration makes it easier to comprehend how the study was conducted and how similar research can be replicated.

It is clarified that the main objective of the research was not to confirm the accuracy of the inertial sensor for measuring trunk kinematics, but rather to use its data to predict the clinical variable Vel. In our case, actual movement denotes the acceleration and angular velocity signal obtained directly by the sensor, while predicted movement denotes the numerical value of the mean trunk velocity (Vel) predicted by machine learning models. As mentioned above, the target variable in our prediction has high clinical relevance, and the validity of the models was measured using statistical values (MAE, MSE, $R^{2}$) rather than comparing them with a reference movement measurement system.

## 3. Results and Discussion

The five regression models were trained to predict the Vel target using the predictor characteristic vectors (per-cycle kinematic descriptors) of each movement (flexion–extension (Rx), rotation (Ry), lateralization (Rz)) and make the target values directly comparable with the Vel measured by the IMU gyroscope. The difference between predicted and measured trunk velocities is measured using performance metrics (MAE, MSE, $R^{2}$), and scatter plots and MAE-range bar plots are used to supplement numerical measures with prediction behavior in the value distribution.

### 3.1. Flexion–Extension

Table 4 shows the performance evaluation result of the five ML models used in flexion–extension prediction. The GradientBoostingRegressor model performed the best with an MAE of 1.11 in cross-validation and also marked the best MAE on the test set (1.12), representing positive generalization to unobserved data. LightGBM showed the minimum test MSE (2.36) and maximum $R^{2}$ (0.99), indicating that almost all variability was described during trunk velocity flexion–extension. GradientBoostingRegressor scored well in validation but was slightly prone to outliers, as indicated by the relatively higher test MSE (6.26). A standard deviation of approximately 1.15 represents a small relative error in absolute motion, suggesting that such error is unlikely to compromise clinical interpretation. This underscores the potential use of LightGBM, which better describes variability, and Gradient Boosting, which better maintains the mean absolute error, as the most balanced and reliable models for tracking flexion–extension in patients with low back pain.

**Figure 3:**
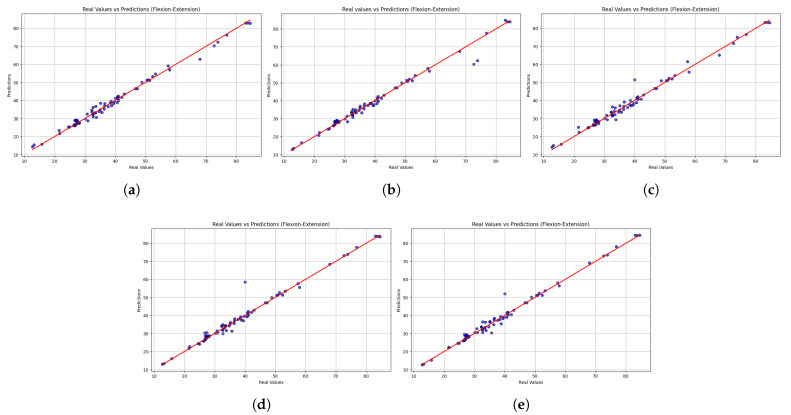
Scatter plots comparing real versus predicted trunk velocity values obtained from the five machine learning models: (**a**) LightGBM, (**b**) XGBoost, (**c**) HistGradientBoostingRegressor, (**d**) GradientBoostingRegressor, and (**e**) StackingRegressor. Each point represents an individual observation, while the red dashed line corresponds to the identity line (perfect prediction). The closer the points are to this line, the higher the accuracy of the model. Results are shown for the flexion–extension task.

Beyond numerical performance metrics, scatter plots provide a visual perspective on model consistency and alignment with actual values. The scatter plots in Figure 3 indicate that all five models were able to predict the observed values of the flexion–extension movements. Overall, the predictive reliability of the models was evidenced by their alignment with the identity line. LightGBM showed the closest alignment with the identity line and the narrowest dispersion, consistent with its lowest MSE and highest $R^{2}$. The second-ranked sequence is XGBoost, which is slightly underperforming at higher speed levels. In comparison, the variability in extreme values was disproportionately high with HistGradientBoostingRegressor and GradientBoostingRegressor and therefore prone to outliers. The dispersion obtained in StackingRegressor was the most dispersed, thus offering the least predictable results. From a rehabilitation perspective, the results indicate that LightGBM offers a more reliable trunk velocity estimate and is the most clinically useful model for monitoring trunk velocity during rehabilitation.

**Figure 4:**
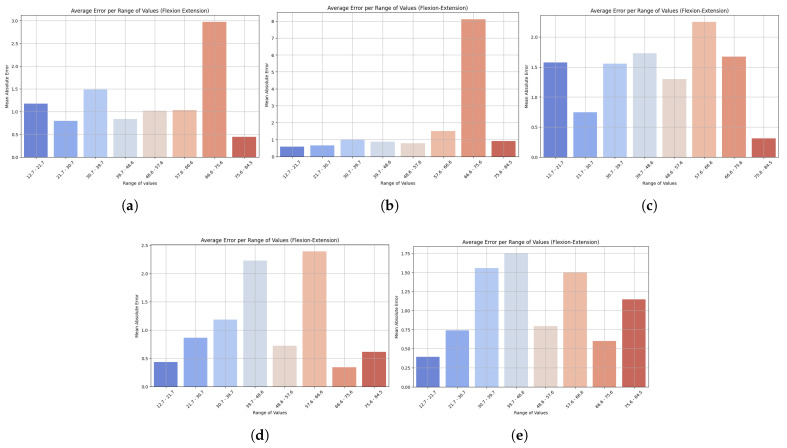
Bar plots show the mean absolute error (MAE) across different ranges of trunk velocity values resulting from the five machine learning models: (**a**) LightGBM, (**b**) XGBoost, (**c**) HistGradientBoostingRegressor, (**d**) GradientBoostingRegressor, and (**e**) StackingRegressor. Lower bars indicate higher accuracy, while higher bars represent higher prediction error at specific motion ranges. This visualization highlights the robustness of the model and its sensitivity to extreme values. Results are presented for the flexion–extension task.

To complement this view, bar charts illustrate how prediction errors vary across different motion ranges, offering insight into the model’s robustness to extreme values. As seen in Figure 4, LightGBM had the lowest and most consistent MAE across all observations, demonstrating robustness and good generalization ability. It also produced small errors at low speeds and relatively small errors at high levels, indicating stability across different motion intensities. XGBoost, on the other hand, was more variable, showing higher variability, particularly due to poor outlier handling. HistGradientBoostingRegressor performed reasonably well and exhibited gradual growth in MAE with higher ranges, while GradientBoostingRegressor had stable performance in the mid-range but increased at the top. The performance of StackingRegressor was similar to that of LightGBM, with greater variability in predictions in the middle of the range of values. These results highlight the potential for clinical application of LightGBM across different movement intensities, making it a reliable tool for monitoring trunk flexion–extension velocity, a key consideration in tracking patient progression.

### 3.2. Rotation

The performance of the models in predicting rotational motion is summarized in Table 5. StackingRegressor performed the best on the test set, with the lowest MAE (0.91) and MSE (1.62) and the best cross-validation result (1.27 MAE). LightGBM scored the best on this metric, as it is the only method obtaining the lowest cross-validation MSE of 3.18 and a very high $R^{2}$ of 0.99 in both validation and testing, an indication of good generalization ability. XGBoost also showed reasonable results with slightly higher error metrics, implying that it is not as robust. In comparison, HistGradientBoostingRegressor and GradientBoostingRegressor were not among the best, and were not leaders in any of the considered metrics. Overall, StackingRegressor performed better on the test data, while LightGBM generalized more easily to new data, so both models could be used for rotational motion prediction. Maintaining prediction errors within 1 degree for trunk rotation demonstrates that the models can provide practically relevant feedback, making them a useful tool for monitoring trunk recovery in post-stroke patients.

**Figure 5:**
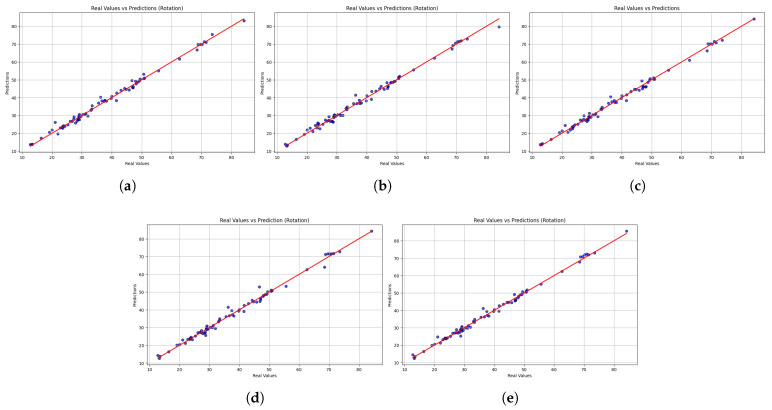
Scatter plots comparing real versus predicted trunk velocity values obtained from the five machine learning models (**a**) LightGBM, (**b**) XGBoost, (**c**) HistGradientBoostingRegressor, (**d**) GradientBoostingRegressor, and (**e**) StackingRegressor. Each point represents an individual observation, while the red dashed line corresponds to the identity line (perfect prediction). The closer the points are to this line, the higher the accuracy of the model. Results are shown for the rotation task.

While the table highlights the overall accuracy, the scatter plots reveal each model’s performance across the entire spectrum of motion values. Figure 5 shows the scatter plot comparing the actual and predicted values of rotational motion. The overall accuracy was high, with most models showing predictions consistent with the line of identity. This reliability is seen with LightGBM, which shows the highest and most consistent predictions, indicated by its high $R^{2}$ value (0.99) and low MSE. XGBoost also performs well, but more errors are observed at high values, decreasing accuracy there. The error of HistGradientBoostingRegressor and GradientBoostingRegressor is larger at the extreme values, with error spikes at the margins, compared to StackingRegressor, which shows very few large anomalies at the extremes. The closeness of LightGBM’s predictions suggests that it can be trusted to provide reliable estimates of trunk rotation velocity and that it can be used to guide customizable rotational exercises in the rehabilitation process. However, the larger errors at the extreme values raise concerns about the possibility of correct recognition in unusual movement patterns.

**Figure 6:**
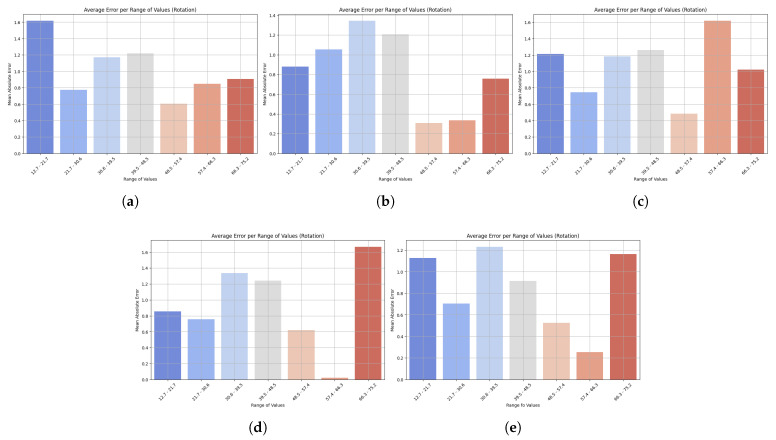
Bar plots show the mean absolute error (MAE) across different ranges of trunk velocity values resulting from five machine learning models: (**a**) LightGBM, (**b**) XGBoost, (**c**) HistGradientBoostingRegressor, (**d**) GradientBoostingRegressor, and (**e**) StackingRegressor. Lower bars indicate higher accuracy, while higher bars represent higher prediction error at specific motion ranges. This visualization highlights the robustness of the model and its sensitivity to extreme values. Results are presented for the rotation task.

Bar plots reveal subtle weaknesses, especially when motions reach atypical ranges, contrasting with the scatterplot results which confirm strong generalization. The distribution of MAEs across various ranges of rotational motion predictions is shown in Figure 6. All models showed low errors at low and medium values, but higher inaccuracies at the extremes. LightGBM also showed stable accuracy across all levels, with only a slight increase in MAE at the highest levels, reflecting strong generalization ability. However, at very high and low values, XGBoost showed a steeper increase in the number of errors, reducing its reliability. HistGradientBoostingRegressor also remained stable at the mid-range, but increased in error at the endpoints, while GradientBoostingRegressor produced fewer overall errors, although its performance was less consistent at the extremes. StackingRegressor performed well at low and medium levels, but had higher errors in extreme cases. LightGBM’s ability to consistently predict trunk rotation across most of the range of motion suggests that it can reliably track rehabilitation activities, with reduced accuracy only at uncommon or extreme ranges.

### 3.3. Lateralization

Table 6 compares the performance of five models in predicting lateralization velocity. It shows that StackingRegressor performed the best overall, with the lowest MAE and MSE in both cross-validation and testing, indicating high accuracy and stability. GradientBoostingRegressor came in second and showed almost the same metrics, indicating its accuracy. XGBoost also performed reasonably well, albeit with higher errors in cross-validation. LightGBM performed worst in MAE and MSE, implying low prediction accuracy despite a good R^2^. Overall, StackingRegressor turned out to be the most accurate and generalizable lateralization model, while LightGBM was the least stable. The low error rates in lateralization prediction highlight the potential for monitoring lateral flexion movements, which are frequently used to assess functional capacity and balance in people with low back pain.

**Figure 7:**
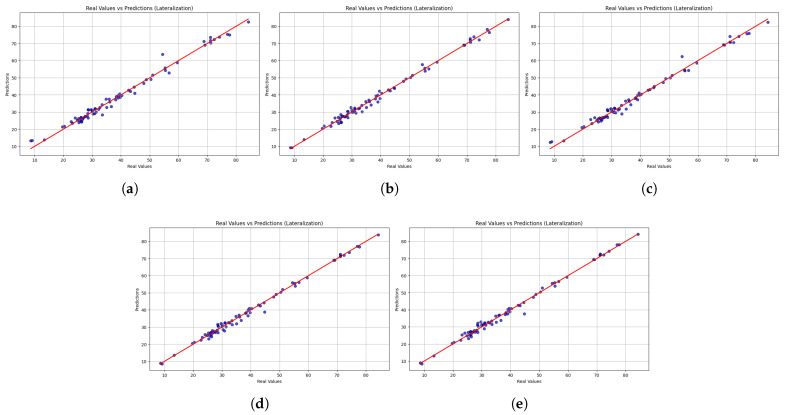
Scatter plots comparing real versus predicted trunk velocity values obtained from the five machine learning models (**a**) LightGBM, (**b**) XGBoost, (**c**) HistGradientBoostingRegressor, (**d**) GradientBoostingRegressor, and (**e**) StackingRegressor. Each point represents an individual observation, while the red dashed line corresponds to the identity line (perfect prediction). The closer the points are to this line, the higher the accuracy of the model. Results are shown for the lateralization task.

In addition to numerical performance measurements, scatter plots provide a qualitative insight into the models’ ability to maintain consistency and align with actual values. Figure 7 shows the scatter plot of the predicted and actual values of lateralization movements. While all models generally followed the line of identity, the accuracy of predictions differed. LightGBM demonstrated stable performance but has significant drawbacks at extreme values, resulting in higher MAE and MSE values. XGBoost demonstrates lower dispersion and high accuracy at lower and upper ranges, while HistGradientBoostingRegressor has a similar trend, albeit with slight deviations at higher values. The Gradient Boosting Regressor had consistent predictive performance with low dispersion, while the Stacking Regressor was the closest to the line of identity compared to the other models, in addition to presenting more accurate overall predictions. The stability of the stacking regressor and the gradient boost regressor along the entire length of the lateralization indicates an enormous potential to correctly predict the velocity of the trunk during lateralization movements, a significant marker of trunk stability and functional recovery. In contrast, the low reliability of LightGBM with respect to outliers could restrict its use, especially in identifying atypical or unusual movement patterns.

**Figure 8:**
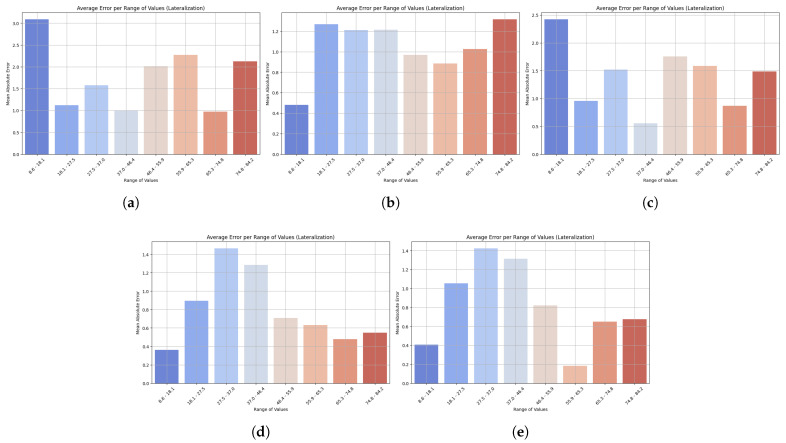
Bar plots show the mean absolute error (MAE) across different ranges of trunk velocity values resulting from the five machine learning models: (**a**) LightGBM, (**b**) XGBoost, (**c**) HistGradientBoostingRegressor, (**d**) GradientBoostingRegressor, and (**e**) StackingRegressor. Lower bars indicate higher accuracy, while higher bars represent higher prediction error at specific motion ranges. This visualization highlights the robustness of the model and its sensitivity to extreme values. Results are presented for the lateralization task.

Figure 8 shows the change in MAE across value ranges in lateralization prediction. Across all models, the error was highest at higher ranges, but the value varied across them. LightGBM showed the highest errors at both the low end (6.8 to 14.1) and the upper end, meaning that its performance is poor at both lower and higher values. The MAE was also high at the upper range for XGBoost, and its counterpart, HistGradientBoostingRegressor, exhibited the same trend with increasing errors at higher values. The GradientBoostingRegressor showed minimal MAE at low and medium levels, with only a controlled increase at the high level, demonstrating good generalization. StackingRegressor performed the best overall, with stable, low MAE at low and medium values, which started to curve upwards at high values. StackingRegressor and GradientBoostingRegressor demonstrated consistent performance across the lateralization spectrum, indicating their suitability for reliable monitoring of lateral flexion velocity during rehabilitation. In contrast, LightGBM’s lower accuracy at the extremes of movement limits its clinical utility.

The exceptionally high $R^{2}$ values observed (up to 0.99) may present a risk of overfitting; however, this potential confounding factor was systematically addressed. For all three cardinal movements, flexion–extension, rotation, and lateralization, our methodology incorporated a robust k-fold cross-validation scheme. Using this method ensured accurate and reproducible separation of the training and test data partitions, justifying the models’ satisfactory performance on a separate subset of the data and confirming the generalization of the learned relationships. Synthetic data amplification was also judiciously employed to reduce the likelihood of model memorization. This was carefully analyzed and implemented to scale the dataset without losing the integrity of the original statistical distributions in the kinematic and kinetic parameters and to be able to work correctly with them. By augmenting the data in a manner that mirrored its inherent structure, the models were compelled to learn the fundamental relationships within the dataset rather than simply memorizing specific instances, which consequently reduced the propensity for overfitting. Despite these robust internal validation strategies, the ultimate generalization of these findings to a broader patient population requires external corroboration. Therefore, external validation of the models using independent clinical datasets is imperative. This final phase of validation is critical to definitively ascertain the models’ capacity to accurately predict mobility outcomes in a new and distinct cohort, thereby confirming their potential for real-world clinical deployment.

### 3.4. Physical and Technical Analysis of the Models

To describe the results in more depth, it is necessary to examine the physical and technical drivers of the models for each type of movement performance. The main movements in an anatomical plane are flexion, extension, and lateralization, which are more biomechanically predictable. In this regard, the use of boosting-based models, such as the GradientBoosting Regressor, proved very effective in addressing nonlinear functional correlations between sensor data and movement speed.

Trunk rotation, on the other hand, is an inherently more complex movement, likely highly biomechanically variable, and in which compensatory movements are common. This complexity can generate more noise in the data and therefore make prediction difficult when using a single model. In this case, the Stacking Regressor performed better than the other models. This method, which utilizes the integration of multiple base models, can be used as a voting system to minimize the effects of individual variability and improve prediction robustness with highly variable and less consistent data, such as rotational movement.

### 3.5. Statistical Analysis

Statistical analysis provided additional insight into the comparative performance of the machine learning models. As shown in Table 7, the ANOVA results demonstrated significant variance between the models in flexion–extension (*p* = 0.0023) and lateralization (*p* = 0.0005), and no significant variance in rotation (*p* = 0.8363). The post hoc HSD test with Tukey showed that GradientBoostingRegressor significantly outperformed the other models in flexion–extension and lateralization tasks, where *p*-values were less than 0.01 in most comparisons within each model. No significant differences were detected in rotational motion, indicating either higher intrinsic variance or reduced sensitivity to visualizing the models through biomechanical patterns. For simplicity and abbreviation, only significant pairwise comparisons indicated by Tukey were listed, without indicating the actual outcome of all pairwise tests. The parametric technique allows the most significant disparities to be highlighted without providing excessive detail that could obscure the overall results. Overall, the findings support the validity of gradient-based ensemble techniques for acquiring movement-specific biomechanical characteristics, as well as the importance of aligning predictive modeling approaches with the nature of the functional movement examined. Overall, the measures of correspondence between performance metrics and statistical comparisons confirm that the GradientBoostingRegressor is the most appropriate and accurate model, especially for flexion–extension and lateralization activities, and that the Stacking Regressor is the most reliable model for rotation.

## 4. Limitations

It is acknowledged that this study has certain limitations that could influence the generalization of the results. The model was trained and validated on a total dataset of 2160 movement samples, which, although a considerable amount, may not be sufficient to capture the full diversity of human trunk biomechanics. A larger and more diverse dataset, particularly with a greater number of samples for each type of movement, could provide more robust statistical power to identify more subtle differences and patterns.

Additionally, the sample of 77 subjects came from a single geographic region in Ecuador and focused on a group of patients receiving a specific therapy. This limits the generalization of our findings to other populations with different demographic characteristics or to patients with low back pain who do not have specific comorbidities or who are not undergoing the same treatment. It is suggested that future studies should include a larger number of participants from different regions to validate the clinical applicability of the predictive models in a broader spectrum of patients with low back pain.

## 5. Conclusions

This study developed and evaluated machine learning models to predict mobility in patients with chronic low back pain using inertial sensors. The results show that GradientBoostingRegressor achieved the best performance in flexion–extension and lateralization, while StackingRegressor excelled in rotation, and LightGBM showed great generalization capabilities across multiple movements. However, no model was clearly superior in all tasks, highlighting the importance of choosing algorithms based on the nature of the movement being analyzed.

Statistical analysis using ANOVA and post hoc Tukey tests confirmed significant differences in flexion–extension and lateralization, providing robustness to the comparison between models and validating the superiority of gradient boosting-based approaches. However, the absence of differences in rotation suggests greater biomechanical variability or lower sensitivity in discriminating between models in this type of movement.

These findings indicate that the combination of low-cost sensors and ML algorithms can become an objective and accessible tool to complement clinical assessment and rehabilitation monitoring in resource-limited settings. Limitations include the small sample size and the need for external validation. Future work should expand the study population, integrate clinical data and medical images, and explore more complex architectures (e.g., deep neural networks) along with mobile applications and wearable devices that enable real-time implementation.

## Figures and Tables

**Table 1:** Summary of studies on ML approaches for LBP patients.

Author/Year	Population	Sensors	ML Approach	Outcome
Kraus, M. et al., 2023 [20]	30 LBP patients	IMUs	Random Forest	Classified LBP vs. healthy with high accuracy
Hartley, T. et al., 2024 [21]	42 adults	Wearable accelerometers	SVM	Identified abnormal trunk movement
Molnar, M. et al., 2018 [22]	25 patients	IMU lumbar region	CNN	Detected mobility impairment levels
Salvatore, T. et al., 2023 [23]	68 subjects	6-axis IMUs	Gradient Boosting	Estimated trunk range of motion
Camargo, J. et al., 2021 [24]	50 participants	Multi-IMU	XGBoost	Classified postural patterns
Pouromran, et al., 2021 [25]	80 patients	Inertial sensors	Ensemble ML	Predicted functional scores in rehabilitation

**Table 2:** Features of MoCap database obtained from the correlation analysis to consider in the study.

Variable	Description
Length	Total length in degrees of the specific movement.
Vel	Mean trunk velocity in degrees/seconds of the specific movement.
MaxRange	Overall range of motion in movement (maximum range of motion plus maximum motion range, depending on the specific movement).
Max and Min	Highest value achieved within the range of motion (Max refers to positive values in sensor, and Min for negative values in inertial sensors, depending on specific movement).
Speed.Max and Speed.Min	Average speed of motion in degrees per second in the movement. (The same relation as Max and Min characteristics).
SpeedUp.Harmony	Average acceleration of motion in degrees per second squared in the movement.

**Table 3:** Hyperparameters of machine learning models used consistently across all movements.

Model	Parameters	Values
LightGBM	n_estimators	500
	learning_rate	0.1
	max_depth	6
	subsample	0.8
	colsample_bytree	0.8
	random_state	42
XGBoost	n_estimators	500
	learning_rate	0.1
	max_depth	6
	subsample	0.8
	colsample_bytree	0.8
	random_state	42
HistGradientBoostingRegressor	max_iter	500
	learning_rate	0.1
	max_depth	6
	random_state	42
	max_bins	255
	l2_regularization	1
GradientBoostingRegressor	max_iter	500
	learning_rate	0.1
	max_depth	6
	subsample	0.8
	random_state	42
StackingRegressor	n_estimators	500
	max_depth	6
	random_state	42

**Table 4:** Comparison of performance metrics for machine learning models in flexion–extension movement using test data and cross-validation metrics evaluated through MAE, MSE, and $R^{2}$.

Model	Test Results				Cross-Validation		
	MAE	MSE	R^2^		MAE	MSE	R^2^
LightGBM	1.17	2.36	0.99		1.3	3.15	0.98
XGBoost	1.15	5.09	0.98		1.26	3.25	0.98
HistGradientBoostingRegressor	1.33	4.19	0.98		1.42	3.7	0.98
GradientBoostingRegressor	1.12	6.26	0.97		1.11	3.17	0.99
StackingRegressor	1.16	3.9	0.98		1.3	3.4	0.98

**Table 5:** Comparison of performance metrics for machine learning models in rotation movement using test data and cross-validation metrics evaluated through MAE, MSE, and $R^{2}$.

Model	Test Results				Cross-Validation		
	MAE	MSE	R^2^		MAE	MSE	R^2^
LightGBM	1.01	1.91	0.99		1.27	3.18	0.99
XGBoost	1.05	1.95	0.99		1.35	3.91	0.98
HistGradientBoostingRegressor	0.97	1.69	0.99		1.29	3.53	0.99
GradientBoostingRegressor	1.02	2.40	0.99		1.21	3.02	0.99
StackingRegressor	0.91	1.62	0.99		1.27	4.0	0.98

**Table 6:** Comparison of performance metrics for machine learning models in lateralization movement using test data and cross-validation metrics evaluated through MAE, MSE, and $R^{2}$.

Model	Test Results				Cross-Validation		
	MAE	MSE	R^2^		MAE	MSE	R^2^
LightGBM	1.47	4.30	0.99		1.40	3.73	0.98
XGBoost	1.13	2.07	0.99		1.19	2.72	0.99
HistGradientBoostingRegressor	1.25	3.25	0.99		1.33	3.29	0.99
GradientBoostingRegressor	1.03	2.05	0.99		1.07	2.02	0.99
StackingRegressor	1.08	2.34	0.99		1.12	2.33	0.99

**Table 7:** *p*-values from one-way ANOVA and Tukey’s HSD post hoc tests across machine learning models for each movement.

Movement	ANOVA (*p*-Value)	Tukey HSD—Significant Comparisons (*p* < 0.05)
Flexion–Extension	0.0023	GradientBoosting vs. LightGBM (*p* = 0.0499); GradientBoosting vs. HistGBR (*p* = 0.0008)
Rotation	0.8363	No significant pairwise differences detected
Lateralization	0.0005	GradientBoosting vs. LightGBM (*p* = 0.0012); GradientBoosting vs. HistGBR (*p* = 0.0101); HistGBR vs. Stacking (*p* = 0.0454); LightGBM vs. Stacking (*p* = 0.0056)

## Data Availability

The original data presented in the study are openly available at https://drive.google.com/drive/folders/19wiB02gjfAum9CAcFRKj_5QgsPfsdD4c?usp=sharing (accessed on 7 October 2025).
